# Understanding Consumers’ Food Waste Reduction Behavior—A Study Based on Extended Norm Activation Theory

**DOI:** 10.3390/ijerph19074187

**Published:** 2022-04-01

**Authors:** Jingjing Wang, Mingyue Li, Sinan Li, Kai Chen

**Affiliations:** School of Economics and Management, Beijing Forestry University, Beijing 100083, China; wangjingj97@bjfu.edu.cn (J.W.); limingyue_2019@bjfu.edu.cn (M.L.); lisinanok666@126.com (S.L.)

**Keywords:** food waste reduction, norm activation model, self-efficacy

## Abstract

Based on norm activation theory, a research framework was built to explore the food waste reduction behavior when consumers eat out. The framework included behavior intentions and four psychological factors: awareness of consequence (persons understanding that actions have consequences), ascription of responsibility (duty to respond), self-efficacy (belief in own skills and capacity), personal norm (individuals’ values to act by socially accepted rules and reduce food waste as a code of conduct and moral obligation). A total of 514 samples from different regions of China were collected through an online survey platform, and the research framework was tested by applying structural equation modeling (SEM). This study found that ascription of responsibility and self-efficacy can effectively activate personal norm to reduce food waste. Personal norm and self-efficacy had a significant positive effect on behavior intentions to reduce food waste. Specifically, self-efficacy had the greatest effect on personal norm, followed by ascription of responsibility, and on behavior intentions to reduce food waste, followed by personal norm. Interestingly, while ascription of responsibility and self-efficacy had an impact on personal norm, awareness of consequence did not significantly influence personal norm to reduce food waste, suggesting that emotional factors are more likely to trigger personal norms that motivate consumers to take action to reduce food waste than cognitive factors. Based on the findings, several suggestions are provided for more effective interventions by restaurants to promote food waste reduction behavior, such as information intervention strategies, displaying information related to food consumption, and reducing the size of plates for some meals.

## 1. Introduction

Humanity faces a grand challenge in determining how to better feed the world’s population on a more crowded planet [1] due to the continuous development of human society. However, increasing competition for the use of water, land, and energy may limit the production of more food [2]. Therefore, it is important to take another promising approach: reduce the amount of food wasted [1]. One study showed that about 1.3 billion tons of food was lost and wasted per year, which accounted for one third of the total food production [3]. Although China feeds 19% of the global population with 7% of the world’s arable land, food waste is still worthy of attention, especially in catering. It was estimated that the total amount of food wasted in China was equivalent to an annual ratio of 300 million people. Among that, households wasted 5.5 million tons of grain, which could feed 15 million people each year and was about 5% of the total amount of food wasted. The food waste caused by cafeterias in all types of schools, enterprises, and institutions could feed 30 million people every year, which is about 10% of the total food waste [4], while the food wasted on the table in restaurants in Chinese catering consumption was equivalent to an annual ratio of 200 million people [5], which is about 66.7% of the total amount. Food waste increases consumers’ expenditure, leads to the abuse and ineffective consumption of labor, water, energy, land, and other resources in the process of food production, and results in the aggravation of environmental pollution [6]. Food waste in the catering industry is not only an environmental problem but also an economic and social problem [7]. Therefore, minimizing food waste in the catering industry is important to reduce the impact of food waste on the environment. The 12.3 sub-goal of the United Nations Sustainable Development Goals clearly states that the global per capita food waste in the retail and consumer sectors will be halved by 2030 [8]. Meanwhile, China is setting up the “Reduce Food Waste—Action in China” platform to raise awareness of food waste reduction and make it easier for stakeholders to have direct access to the food waste reduction network. Scholars have carried out numerous studies on food waste from different perspectives but have not yet formed a unified definition [9]. One of the definitions states that food waste is the food used for consumption or processing that is discarded at the food retail stage or final consumption [3]. Eating out as a common occurrence means patronizing both street vendors and various dining outlets [10]. One study referred to eating in a restaurant specifically, including casual and fast casual dining. Therefore, in the context of restaurants, according to the characteristics of eating out and the definition of food waste [3], the research defined food waste reduction when eating out as ordering food as needed to avoid unnecessary waste, cherishing all sorts of food to reduce plate waste and packing leftovers, and food waste as the loss of food that could have been avoided under existing conditions due to people’s irrational consumption purposes and behaviors.

As a worldwide problem, scholars have carried out numerous studies on food waste from different perspectives. Graham-Rowe argued that individuals will not reduce food waste unless they are motivated to do so [11], so it is important to explore the influence factors of food waste. Previous studies mainly explored the impact of the economy [12], culture [13], demographic characteristics [14], and psychological determinants [15] on household or restaurant food waste. Among these studies, most scholars explored the influencing factors of food waste by taking the family [11] as the research object and analyzed the influence of income, knowledge [16], packing [17], family composition, family size [18], shopping habits [19], and other factors on household food waste behavior. Most of the existing studies analyzed the influence factors of food waste at the household level rather than in the context of restaurants [15,20]. However, most food waste occurring at the consumer end of the chain comes from restaurants, and it is more common in developing countries due to the increase in eating out [21]. Yang et al. noted that the food waste generated by commercial restaurants accounted for 62% of the total food waste at the consumer end of the chain in China [22]. Many scholars also pointed out that the food service sector was responsible for about half of all food waste in the Chinese food supply chain [23]. Thus, the issue of restaurant food waste should be a concern of academics and the public. Several studies explored restaurateurs’ attitudes and behaviors toward food waste [24], such as overproducing and food donation, rather than those of the consumers. Meanwhile, Lorenz et al. discussed the reason why people leave their food in the canteen based on the theory of planned behavior [25], and Sirieix et al. explored consumers’ attitudes towards doggy bags and the obstacles to promote doggy bags and reduce food waste [26]. Few studies have explored the psychological factors concerning the behavior towards food waste when eating out based on norm activation theory. However, interventions may be effective when designed to target the key psychological factors that underpin motivations for food waste reduction [11]. Therefore, understanding the psychological factors of consumers’ food waste behavior and discussing how to avoid waste more effectively are key to reducing food waste.

Norm activation theory is also known as the norm activation model (NAM); it was first proposed by Schwartz [27], and then it was widely used to predict pro-social behavior and to investigate environmental protection intention and behavior [28]. The NAM is composed of awareness of consequence (AC), ascription of responsibility (AR), personal norm (PN), and a person’s pro-environment behavior. The key point of the model is that activation of the personal moral norm will affect the occurrence of individual behavior [29]. Many scholars have carried out research on environmental protection behaviors by drawing upon the norm activation model [30,31]. With the development of research, several scholars expanded the model and obtained better results, such as Shin et al., who integrated the theory of planned behavior and the NAM to explore consumers’ intention to choose featured organic dishes at restaurants and proved the proposed model was applicable [32], as well as Klöckner and Blöbaum, who integrated the theory of planned behavior, the NAM, the theoretical concept of habit, and ipsative theory to explore the travel mode choice and proved the comprehensive model explained the greatest degree of variation compared with the other models [33]. It can be seen that the original NAM is not appropriate for every study, and some adjustments should be made to better explain specific environmentally friendly behaviors.

As a result of the above analysis, this study took the food waste reduction behavior when eating out as the research object, based on the context of eating out, and introduced the variable of self-efficacy to extend the norm activation model. Then, this study obtained data by questionnaires and applied structural equation modeling (SEM), which was used to evaluate the adequacy of the proposed framework and test the hypothesized relationships among the study constructs [29], in order to explore the influence of psychological consciousness on food waste reduction behavior. Structural equation modeling (SEM) is a statistical method to analyze the relationship between variables based on the covariance matrix of the variables and can deal with both latent variables and their indicators. It is noteworthy that, according to the theory of planned behavior, people with stronger intentions tend to engage in a certain behavior [34], so this study indicated and predicted the real behavior through behavior intentions. This study will enrich the application context of the norm activation model in the field of sustainable consumption, as well as providing suggestions for catering enterprises to better intervene in consumers’ food waste reduction behavior and promote social sustainable development.

The unique features of this study are mainly the following two aspects. One is the innovation of the research context. Previous studies on food waste reduction behavior were mostly conducted in the context of family life, and most of the food waste occurred in restaurants [21], so this study extended the research context to eating out to explore the factors that influence consumers’ behavior intentions to reduce food waste and their mechanisms of action in the eating out context. Another innovation is the addition of self-efficacy as a psychological factor based on the norm activation model (NAM), which helps to construct a more comprehensive theoretical framework of environmental psychology and can better explain consumers’ food waste reduction behavior, extending the application of the norm activation model in environmentally friendly behavior.

## 2. Literature Review and Hypothesis Development

### 2.1. Personal Norm

Personal norm is defined as the moral responsibility of individuals for specific actions, and it refers to “the moral obligations to perform pro-social behaviors” [27], which relate to the individual’s internalized values [35]. Many studies showed that personal norms have a direct impact on individuals’ environmentally friendly behaviors [31,32,36]; personal norms are even the trigger of individuals’ pro-environment intentions [37]. The behaviors to comply with personal norms are not based on the fear of social sanctions, but on avoiding negative emotional experiences, such as guilt, regret, and shame [38]. The personal norm of saving food concerns people reducing food waste as a code of conduct and moral obligation. Wasting food and not complying with norms can make consumers feel shame or guilt [39], which will be regarded as a type of self-sanction by themselves. Therefore, they are more likely to reduce food waste in order to strive for positive emotions and avoid negative emotions [40]. To sum up, the following hypothesis is proposed:

**H1.**  *Personal norm will have a positive impact on behavior intentions to reduce food waste and cause an individual to act in a way that reduces food waste*.

### 2.2. Antecedents of Personal Norm

Awareness of consequence is an individual’s cognition of the negative consequences when they do not act pro-socially [36]. Reducing food waste is a type of environmentally friendly behavior that has many positive environmental and socio-economic ramifications. Therefore, awareness of food waste consequences refers to consumers’ cognition of the negative effects caused by not saving food. Research has shown that awareness of consequence can directly affect personal norm [41]. Ascription of responsibility means the individuals’ feelings of responsibility for the negative consequences caused by not performing environmentally friendly behaviors [36]. That is, when people believe that the responsibility is ascribed to themselves, they will tend to engage in waste reduction behavior [42]. Wang’s study showed that when tourists deem that they should be responsible for the negative consequences of waste generated by themselves at tourist destinations, they will be more likely to feel the moral obligation to reduce waste [43]. Judith et al. conducted many studies about residents’ acceptance of energy policies, citizens’ behavior of blood donation, etc. The research results showed that ascription of responsibility had a significant impact on personal norm [31]. As for food waste, when consumers consider the negative consequences of food waste, such as the serious environmental and social problems, they will come up with ideas for things they need to do to reduce the negative consequences, promoting the formation of food-saving personal norms [44]. Reducing food waste is the responsibility of the public, and everyone is responsible for the negative consequences of food waste. This type of responsibility will activate the personal norm of consumers to save food. To sum up, the following hypotheses are proposed:

**H2.**  *Awareness of the consequence of food waste will affect personal norm and cause an individual to act in a way that reduces food waste*.

**H3.**  *Ascription of responsibility (duty to respond) of food waste will affect personal norm and cause an individual to act in a way that reduces food waste*.

Self-efficacy is defined as the degree to which individuals believe they are capable of accomplishing something [45]. In a specific context, such a belief enables individuals to generate relevant motivations and to take a series of actions to accomplish a specific task [45]. Self-efficacy is not based on individuals’ real ability, but on their evaluation of their own ability [46]. In this study, self-efficacy refers to individuals’ belief and confidence in their capability to take a series of actions to reduce food waste effectively and alleviate existing environmental problems.

Consumers will measure whether they can improve some environmental problems by saving food, as well as whether they have the ability to influence others to reduce food waste when eating out. Positive evaluation results will stimulate consumers’ responsibility to save food; when individuals believe that their action is better for the environment, they will internalize their own responsibility consciousness into daily consumption behavior and avoid the guilt of not implementing.

At the same time, the results of the evaluation will adjust the level of consumers’ efforts, the choice of behavior, and the performance in a specific task [47]. That is, the level of self-efficacy affects the decision of whether an individual carries out a certain behavior [34]. Several studies have shown that self-efficacy was related to people’s pro-social behaviors [48,49]. When consumers believe that their food-saving behavior is helpful to improve the environment, the action of consumers to reduce food waste will be promoted. The higher the self-efficacy, the more likely it is to reduce food waste. People will form positive attitudes to do something toward behaviors that they believe produce desirable outcomes [50]. Therefore, the following hypotheses are proposed:

**H4.**  *Self-efficacy (believing in one’s own capability) will have a positive impact on personal norm to reduce food waste*.

**H5.**  *Self-efficacy (believing in one’s own capability) will have a positive impact on behavior intentions to reduce food waste*.

**Figure 1:**
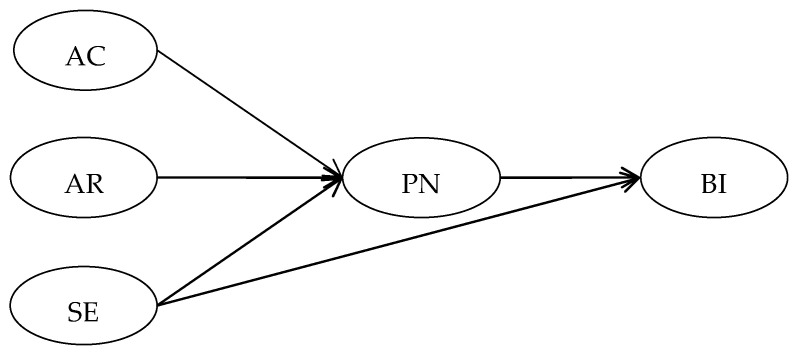
Framework. Note: AC, awareness of consequence of food waste; AR, ascription of responsibility for food waste; PN, personal norm to reduce food waste; SE, self-efficacy; BI, behavior intentions to reduce food waste.

The norm activation model has strong practicability. In the process of application, due to the change in the situation and the difference in research problems, norm activation theory should be adjusted to improve the explanatory power. This study introduced the variable of self-efficacy and established the relation between self-efficacy and behavior intentions on the basis of the norm activation model to analyze the influence of the psychological factors on behavior intentions. The final conceptual model is shown in Figure 1.

## 3. Materials and Methods

### 3.1. Questionnaire Design

The questionnaire was divided into two parts. The first part was the collection of basic information, including respondents’ gender, age, total monthly disposable income, frequency of eating out, and the average cost per meal when eating out. The second was the measurement of the awareness of consequence of food waste, ascription of responsibility for food waste, personal norm to reduce food waste, self-efficacy, and food-saving behavior intentions, which requested the respondents to recall their food waste behavior when eating out in the last month and judge each sentence combined with their actual feelings.

### 3.2. Scale Design

The draft of the questionnaire was formed based on the related literature. Then, we modified the measurement items of all variables combined with the language habits of Chinese people to adapt to the research background in China. After that, discussions were held with scholars to further modify the wording to ensure the content validity of the questionnaire, and then the final draft was formed. For example, according to the definition of reducing food waste by eating out, the item “I should pack the leftovers” was added to “personal norm to reduce food waste”. This study used a five-point Likert scale to test five variables, which ranged from “very disagreed” to “very agree”, assigned from 1 to 5. Some of the measurement items were calculated by reverse assignment, which meant some questions had opposite meanings to others in the scale, and it was necessary to assign from “strongly disagreed” to “strongly agree”, with values of 5, 4, 3, 2, and 1. The specific design of the testing items is shown in Table 1.

### 3.3. Data Collection

From April to May 2019, questionnaires were distributed and collected via “wjx.cn”, an authoritative online survey platform in China, to obtain data from different regions of China. A total of 548 samples were collected. Among them, 34 samples took less than 20 s to answer the questions, which was a significantly shorter amount of time than the others, and the average response time of each question was less than 1 s, meaning that the samples were regarded as invalid samples, leaving 514 valid samples. 

Descriptive statistical analysis of the recovered samples obtained the following results, which are shown in Table 2: there were 253 male respondents (49.2%) and 261 female respondents (50.8%); the proportions of males and females were basically balanced, compared with the Chinese population, where 51.13% of the population were male, and 48.87% were female in 2018. The age group of 18–25 years old (40.5%) and the 30–50-year-old middle-aged group (26.9%) made up the main proportion of the respondents. The respondents were mainly young people and middle-aged people, and among the general population, the former’s education level was mostly undergraduate, while the latter’s education level was mainly college and below. In addition, the respondents had different degrees of differences in the total monthly disposable income, frequency of eating out, and consumption level. The basic characteristics of the samples are representative to some extent.

### 3.4. Model Construction

**Figure 2:**
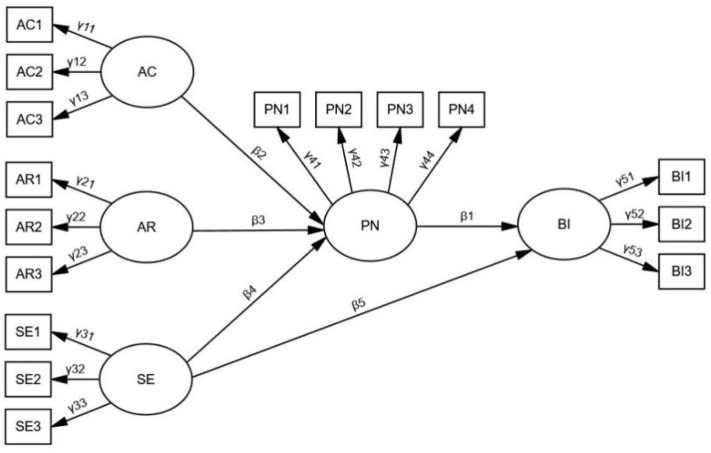
Model. Note: The “ellipse” indicates a latent variable; the “rectangle” indicates an observable variable; and the “single arrow” indicates a causal relationship.

Based on the structural relationship of the research hypotheses of consumers’ behavior intentions to reduce food waste, this study constructed a structural equation model of the factors influencing behavior intentions (Figure 2), with the following mathematical expressions:

AC = γ_11_AC_1_ + γ_12_ AC_2_ + γ_13_AC_3_ + ξ_1_

AR = γ_21_AR_1_ + γ_22_AR_2_ + γ_23_AR_3_ + ξ_2_

SE = γ_31_SE_1_ + γ_32_SE_2_ + γ_33_SE_3_ + ξ_3_

PN = β_2_AC + β_3_AR + β_4_SE + γ_41_PN_1_ + γ_42_PN_2_ + γ_43_PN_3_ + γ_44_PN_4_ + ξ_4_

BI = β_1_PN + β_5_SE + γ_51_BI_1_ + γ_52_BI_2_ + γ_53_BI_3_ + ξ_5_

In the above mathematical expressions, AC_1_, AC_2_, and AC_3_ denote the observable variable of awareness of consequence; AR_1_, AR_2_, and AR_3_ denote the observable variable of ascription of responsibility; SE_1_, SE_2_, and SE_3_ denote the observable variable of self-efficacy; PN_1_, PN_2_, PN_3_, and PN_4_ denote the observable variable of personal norm; BI_1_, BI_2_, and BI_3_ denote the observable variable of behavior intentions; β is the path coefficient between latent variables; γ is the loading coefficient between observable and latent variables; ξ is the residual term.

## 4. Results

### 4.1. Reliability and Validity Tests

The AMOS23.0 software was used to conduct confirmatory factor analysis, and then the goodness of fit of the model was calculated. Regarding the absolute goodness of fit indexes, the ratio of chi-square to degrees of freedom (X^2^/DF) was 3.115, less than the reference value of 5, and the root mean square error of approximation (RMSEA) was 0.064, less than the reference value of 0.08. Meanwhile, all of the value-added goodness of fit indexes, such as the goodness of fit index (GFI), adjusted goodness of fit index (AGFI), relative fitting index (RFI), incremental fit index (IFI), and comparative fit index (CFI), were greater than the reference value of 0.90, indicating that the overall fitting degree of the model was reliable.

Then, the SPSS22.0 software was used to test the reliability of the total scale and each variable; the test results are shown in Table 3. Cronbach’s α and the composite reliability value (CR value) of each variable were greater than 0.7 [54], indicating that the overall reliability of the data used in this study was good and the scale of this measurement had high reliability.

The scale was also tested for structural validity by AMOS23.0. Convergence validity was tested, and the result is shown in Table 2. It was found that the composite reliability (CR) value for each variable was greater than the minimum of 0.7 [55], and the average variance extracted (AVE) value of all the variables also exceeded the minimum of 0.5 [56], which indicates that the questionnaire had good convergence validity [57]. 

Then, the study conducted a discriminant validity test by comparing the AVE arithmetic square root of each variable and the correlation coefficient between each variable, and the results show that the arithmetic square roots of the AVE of most variables were greater than the corresponding interface correlation coefficient.

### 4.2. The Goodness of Model Fit Test

The results of the goodness of fit of the model are shown in Table 4. Table 4 shows that the GFI, AGFI, NFI, and CFI values were, respectively, 0.966, 0.911, 0.951, and 0.966, all of them being greater than 0.9 [58], which is the threshold condition of the fitness test standard. Additionally, the RMSEA was 0.063, less than the reference value of 0.08. A total of 12 indicators, as shown in Table 3, including the absolute goodness of fit index, the value-added goodness of fit index, and the simplified goodness of fit index, were all in accordance with the fitness test standard, which meant the model had a good fitting effect, and the theoretical model proposed in this study was consistent with the actual research data.

### 4.3. Hypothesis Testing

This study used AMOS23.0 to establish the SEM model and verify the relationship between the variables. The path coefficient and the hypothesis test results are shown in Table 5. The CR values of the hypotheses were all greater than the reference value of 1.96, except for H2, which indicates that hypotheses H1, H3, H4, and H5 were confirmed while H2 was not. Moreover, the model explained 63% of the variance in behavior intentions to reduce food waste. The standardized path coefficient between personal norm to reduce food waste and behavior intentions of food saving was 0.254, and between self-efficacy and behavior intentions of food saving was 0.556, which indicates that both personal norm to reduce food waste and self-efficacy had a positive impact on behavior intentions of food saving. Similarly, it can be found that the standardized path coefficients of ascription of responsibility for food waste and self-efficacy in relation to personal norm to reduce food waste were, respectively, 0.246 and 0.735, both of which were significant at *p* < 0.001; this also indicates that self-efficacy had a significant positive effect on personal norm to reduce food waste compared to ascription of responsibility. However, awareness of consequence had no significant positive effect on the food-saving personal norm as expected (*p* = 0.368). These three variables explained 83% of the variation in personal norm to reduce food waste. Moreover, awareness of consequence explained above 80% of the two items, and self-efficacy explained above 50% of the three items, while ascription of responsibility explained above 35% of the two items.

Furthermore, the direct effects, indirect effects, and total effects of standardization between the observable variables were available. The results show that the direct impact of self-efficacy on the behavior intentions of food saving (0.556) was stronger than the indirect impact (0.186). The effect of self-efficacy on behavior intentions to reduce food waste (0.556) was greater than the impact of personal norm to reduce food waste on behavior intentions to reduce food waste (0.254). 

**Figure 3:**
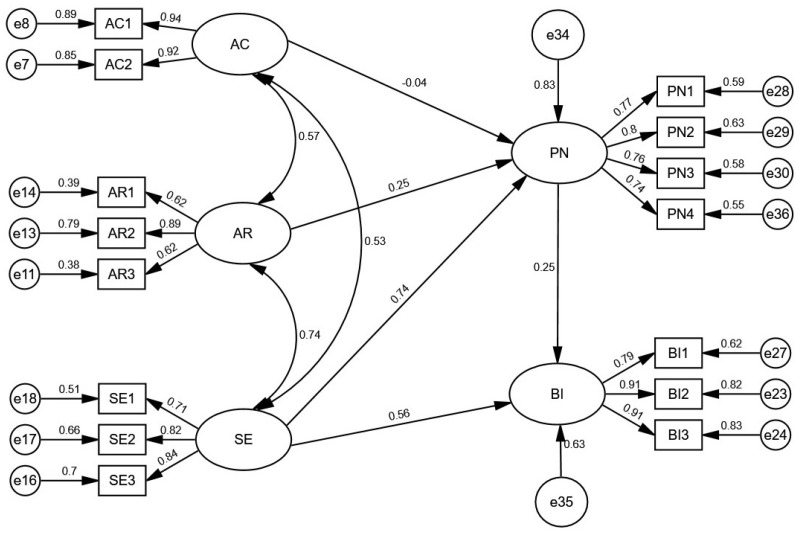
Structural equation model and standardized path coefficient graph. Note 1: AC, awareness of consequence of food waste; AR, ascription of responsibility for food waste; PN, personal norm to reduce food waste; SE, self-efficacy; BI, behavior intentions to reduce food waste. Note 2: The ellipse on the inside represents latent variables; the rectangle represents items of variables; the circle on the outside represents the residual; the number on the line represents the standardized path coefficient; and the number next to the figure represents the explained variances.

The results of the final structural equation model are shown in Figure 3. According to the results of the hypothesis testing, after removing the path that failed the test and keeping the paths that passed the test, three causal chains are clarified in Figure 3: Ascription of responsibility for food waste → Personal norm to reduce food waste → Behavior intentions to reduce food waste, Self-efficacy → Personal norm to reduce food waste → Behavior intentions to reduce food waste, and Self-efficacy → Behavior intentions to reduce food waste. Generally, a larger path coefficient indicates a stronger degree of association, and it can be seen through Figure 3 that the path coefficient of self-efficacy to personal norm is the largest at 0.74, indicating that self-efficacy has the most significant effect on personal norm.

## 5. Discussion and Conclusions

### 5.1. Result Discussion

Based on the norm activation model (NAM), this study introduced self-efficacy as a dependent variable to construct a research framework to explore the influence of psychological factors on consumers’ behavior intentions to reduce food waste in the scenario of eating out. This study found that the norm activation model (NAM) is an effective theory to explain behavior intentions to reduce food waste when eating out. It shows that the independent variable of self-efficacy not only indirectly influences individuals’ behavior intentions through the personal norm to reduce food waste but also has a direct positive influence on individuals’ behavior intentions. This study also enriches the research on food waste reduction and consumers’ environmentally friendly behaviors from the perspective of eating out and provides new ideas for practitioners to implement effective interventions to promote food waste reduction behaviors, such as managers of restaurants.

Influencing factors have different effects on consumers’ behavior intentions. Personal norm has a positive impact on behavior intentions to reduce food waste, which is consistent with existing research findings [29,36]. This is because individuals usually generate a personal norm to avoid negative emotional experiences such as guilt, regret, and shame. If consumers generate a personal norm for saving food, it brings them pride and self-esteem enhancement and satisfaction, and thus individuals are more inclined to reduce food waste to obtain such positive emotions [40]. The stronger the consumers’ personal norm about saving food, the more likely they are to develop behavior intentions to reduce food waste. Consumers’ sense of moral obligation to conserve food keeps them away from feelings of guilt or shame that arise from not conserving food.

The ascription of responsibility exerts an impact on personal norms, which, in turn, motivates individuals to take action to reduce food waste. Specifically, the greater the sense of personal responsibility to reduce food waste when eating out, the more likely it is to motivate consumers to take specific actions of moral obligation. The results validate the relationship between the ascription of responsibility and personal norm to reduce food waste; the personal norm to save food is activated when individuals are aware that their food waste has negative consequences for others and society, and they believe they are responsible for the consequences of these actions [31,44].

In addition, self-efficacy can have both an effect on behavior intentions to reduce food waste by influencing personal norms, and a direct effect on behavior intentions to reduce food waste. On the one hand, when individuals believe that they are capable of reducing food waste and that their behavior contributes to improving environmental problems, the moral obligation that they should act to avoid the guilt of inaction is internalized, and the personal norm to reduce food waste is activated. On the other hand, the higher the self-efficacy, the more likely consumers are to believe that they can improve or solve some of their environmental problems by reducing food waste and have the ability to influence others to motivate them to reduce food waste, and therefore the stronger their behavior intentions to save food when they eat out. Therefore, it is necessary to focus on the content of the guidelines, and the effectiveness of the information intervention will also depend on whether the promotion is effective for consumers.

Finally, it is important to note that awareness of consequence of food waste had no significant effect on personal norm to reduce food waste, which is inconsistent with the theoretical hypothesis. The possible reason for this is that consumers tend to deviate from their perceptions and behaviors when engaging in environmentally friendly behaviors [59]. In recent years, several studies have shown that there is no significant correlation between the level of environmental cognition and environmentally friendly behaviors. Kanchanapibul et al. investigated the green purchasing behavior of the younger generation and pointed out that personal emotional responses are a key motivation for genuine engagement in green issues [60]. Compared to cognitive factors, emotional factors are more likely to trigger personal norms to reduce food waste and have a more important influence and explanatory power on environmentally friendly behaviors.

### 5.2. Management Implications

This study shows that consumers’ ascription of responsibility and self-efficacy will activate their personal norm of food waste reduction and affect their behavior intentions. According to the results, this research provides practical implications for catering enterprises about how to influence consumers’ behavior intentions of food waste reduction for better management.

Catering enterprises can inspire consumers to save food by implementing information intervention strategies, cultivating a sense of responsibility to reduce food waste, and inspiring consumers to save food. For example, restaurants can place the food-saving guidelines on the table prominently to emphasize the importance of food and the necessity to reduce food waste.

Catering enterprises can take the initiative to display information related to food consumption, especially for first-time consumers. Restaurants can request the waiters to inform customers about the size of selected dishes and the information about special ingredients, so as to avoid food waste caused by information asymmetry. At the same time, catering enterprises can ask the waiters to remind consumers to order food as needed and pack the leftovers after the meal.

Reducing the size of plates for some meals represents another strategy, so as to meet the desire of consumers who want to try more dishes. In addition, catering enterprises should pack leftovers for consumers for free, so as to reduce perceived difficulties of food waste reduction for consumers.

Suggestions where catering enterprises come into play have already been mentioned, but further than that, we also provide some actionable suggestions for other stakeholders as follows.

NGOs can initiate social discussions to promote consumers’ reflection on consequences. Meanwhile, NGOs can launch information campaigns to stimulate consumers’ moral obligation, such as communication about irresponsible behavior when ordering food that causes a lot of food waste.

Policymakers can guide consumers to save food by putting up signs to reduce food waste at bus stops, in newspapers, on television, on the radio, and on new media platforms on the Internet, which aim to send messages such as “Saving food is everyone’s responsibility”, and “Reducing food waste is helping to improve environmental problems.” These may deter consumers from wasting food.

Consumers can take the initiative to know the size of the plate and the amount of food, get into the habit of ordering in moderation and packing leftovers, access the information of food nutrition to develop a balanced meal behavior, and fully consider the possible impact of their own behavior on the environment in the process of consumption to maximize long-term environmental benefits.

### 5.3. Limitations and Future Research

Although this research was in-depth, there are still some issues that are worthy of further exploration in the future. Firstly, in this study, the real waste behaviors of consumers when eating out were not measured due to objective conditions. Future studies can explore the relationship between intentions and behaviors based on actually measuring consumers’ food waste behaviors. Secondly, this study only discussed the influence of the NAM on consumers’ food-saving intentions when eating out, and future studies should explore more possible influences to improve the research framework. Third, the research conclusions were based on the survey data of 514 respondents online in China. In future studies, the sample range can be expanded, and further studies on food waste when eating out can be carried out in different contexts, such as cafeterias and restaurants near tourist attractions.

### 5.4. Conclusions

In this study, an SEM model was established which represents an extension of the NAM and verified the relationship between the variables based on 514 valid samples. More specifically, this study explored the effects of awareness of consequence, ascription of responsibility, and self-efficacy on personal norm, and the effects of personal norm and self-efficacy on behavior intentions to reduce food waste in the scenario of eating out. Three main conclusions were drawn from this study.

Firstly, ascription of responsibility and self-efficacy had a significant positive effect on personal norm to reduce food waste. According to Figure 3, the effect of self-efficacy on personal norm to reduce food waste (0.74) was greater than the effect of ascription of responsibility on personal norm (0.25). More specifically, when consumers realize that their food waste behaviors will cause negative impacts on society and the environment, they usually consider themselves responsible for the consequences of these behaviors, and they will activate their personal norms to restrain and limit themselves at the moral level to make themselves reduce food waste behaviors. As for self-efficacy, consumers believe that they have the ability to reduce food waste, that their behavior helps alleviate the deteriorating environmental problems, and that they can motivate others to reduce food waste. By viewing it as their obligation to reduce food waste, consumers activate personal norms to avoid shame, guilt, or regret.

Secondly, we found that personal norm and self-efficacy had a significant positive effect on behavior intentions to reduce food waste. According to Figure 3, the effect of self-efficacy on behavior intentions to reduce food waste (0.56) was greater than the effect of personal norm on behavior intentions to reduce food waste (0.25). More specifically, when consumers feel a sense of moral responsibility and obligation, they are likely to generate behavior intentions to reduce food waste. Consumers believe that reducing food waste is something that must be done, and that they will feel guilty if they do not do it, thus creating constraints on their own behavior. In addition, self-efficacy can influence behavior intentions to save food not only indirectly through personal norm, but also directly through behavior intentions to reduce food waste. When consumers are aware that their self-conduct can play a positive role in reducing food waste, they will take action. 

Thirdly, we found no significant effect of awareness of consequence on personal norm to reduce food waste, which is due to the fact that consumers are often prone to inconsistency between cognition and behavior. Several studies have found that consumers’ cognition is not necessarily effective in influencing environmentally friendly behavior [16], while consumers’ emotions are more effective in motivating environmentally friendly behavior [60]. Compared to cognitive factors, emotional factors are more likely to motivate personal norms and are more likely to have an impact on willingness to behave in an environmentally friendly manner.

This study explored the psychological factors and influences of consumers when dining out and discussed the mechanisms of these factors on behavior intentions to reduce food waste. This study enriches the background of the application of the norm activation model in the field of sustainable consumption and helps restaurant companies to better intervene in consumers’ food waste reduction behavior.

## Figures and Tables

**Table 1:** Measurement items of latent variables.

Latent Variables	Items	Interview Questions	Source
Awareness of consequence of food waste (AC)	AC1	Food waste will cause loss of resources such as fresh water and oil.	De Groot 2009 [31]
	AC2	Food waste will accelerate resource depletion.	
Ascription of responsibility for food waste (AR)	* AR1	Reducing food waste and solving environmental problems is the responsibility of governments and enterprises mainly.	De Groot 2009 [31]
	AR2	I have shared responsibilities to waste of resources and exhaustion of energy.	
	* AR3	Wasting food is a personal choice, is nothing to do with responsibility.	
Personal norm to reduce food waste (PN)	PN1	I have obligations to reduce food waste.	Ellen 2015 [51]
	PN2	I should pack the leftovers.	
	PN3	I have obligations to discourage companions from wasting food.	
	PN4	Many leftovers make me feel guilty.	
Self-efficacy (SE)	SE1	Saving food when eat out is easy for me.	Sun 2012 [52], Sherer 1982 [53]
	SE2	If I don’t waste food, I can promote people to reduce food waste.	
	SE3	I reduce food waste when I eat out is helpful to improve environmental problems.	
Behavior intentions to reduce food waste (BI)	BI1	I will actively discuss with others about how to reduce food waste.	Sun 2012 [52]
	BI2	I will reduce food waste and encourage companions to avoid food waste.	
	BI3	I will explain companions the importance of reducing food waste proactively.	

Note: testing items marked by * were reverse test items, which had opposite meanings to others and were calculated by reverse assignment.

**Table 2:** Sample characteristics.

Gender		Age	
Male	49.2%	17−	6.8%
Female	50.8%	18–25	40.5%
**Level of Education**		26–29	15.0%
Junior college and below	37%	30–50	26.9%
Undergraduate	51.4%	51+	10.8%
Master degree or above	11.6%	**Frequency of Eating Out**	
**Average Consumption per Meal (￥)**		1–3 times a month	38.3%
0–50	14.6%	4–8 times a month	38.9%
51–100	39.5%	9–14 times a month	14.8%
101–200	40.1%	More than 15 times a month	7.8%
201+	5.6%	Never	0%

**Table 3:** Reliability analysis and convergence efficiency analysis results.

Latent Variable	Items	Factor Loading Value	Cronbach’s α	CR	AVE	Arithmetic Square Root of AVE
Awareness of consequence of food waste (AC)	AC1	0.943	0.929	0.930	0.869	0.932
	AC2	0.921				
Ascription of responsibility for food waste (AR)	AR1	0.622	0.762	0.759	0.520	0.721
	AR2	0.891				
	AR3	0.615				
Personal norm to reduce food waste (PN)	PN1	0.768	0.850	0.852	0.590	0.768
	PN2	0.796				
	PN3	0.763				
	PN4	0.744				
Self-efficacy (SE)	SE1	0.716	0.831	0.833	0.626	0.791
	SE2	0.816				
	SE3	0.836				
Behavior intentions to reduce food waste (BI)	BI1	0.788	0.900	0.904	0.760	0.872
	BI2	0.908				
	BI3	0.913				

Note: Factor loading value represents the load of the No. i variable on the No. j common factor; Cronbach’s α is an index of reliability, which measures the internal consistency of the test according to a certain formula; CR is composite reliability, the reliability of a composite score; AVE is the average variance extracted, a statistic that tests the internal consistency of structural variables.

**Table 4:** Result of model goodness of fit.

Fitness Index	Statistical Test Indicators	Fitting Effect	Criteria for Judging	Test Results
Absolute Goodness of Fit Index	X^2^/DF	3.056	<5	Accept
	GFI	0.966	>0.9	Accept
	AGFI	0.911	>0.9	Accept
	RMSEA	0.063	<0.08	Accept
Value-Added Goodness of Fit Index	NFI	0.951	>0.9	Accept
	RFI	0.937	>0.9	Accept
	IFI	0.967	>0.9	Accept
	TLI	0.957	>0.9	Accept
	CFI	0.966	>0.9	Accept
Simplified Goodness of Fit Index	PCFI	0.755	>0.5	Accept
	PNFI	0.743	>0.5	Accept
	CAIC	525.765 < 1376.022 812.872 < 6863.620	Theoretical models are smaller than both saturation models and independent models	Accept

Note 1: X^2^/DF, GFI, AGFI, RMSEA, NFI, RFI, IFI, TLI, CFI, PGFI, PNFI, and CAIC mean the ratio of chi-square to degrees of freedom, goodness of fit index, adjusted goodness of fit index, root mean square error of approximation, normed fit index, relative fitting index, incremental fit index, non-normed fit index, comparative fit index, parsimony goodness of fit index, parsimony-adjusted NFI, and consistent Akaike information criterion, respectively. Note 2: The confidence interval for RMSEA values at a 90% confidence level was from 0.054 to 0.072.

**Table 5:** Results of SEM model test.

Hypothesis		Standardized Coefficient	CR Value	*p*	Results
H1	Personal norm to reduce food waste → Behavior intentions to reduce food waste	0.254	4.102	*	Accept
H2	Awareness of consequence of food waste → Personal norm to reduce food waste	−0.037	−0.900	0.368	Reject
H3	Ascription of responsibility for food waste→ Personal norm to reduce food waste	0.246	4.864	***	Accept
H4	Self-efficacy → Personal norm to reduce food waste	0.735	8.139	***	Accept
H5	Self-efficacy → Behavior intentions to reduce food waste	0.556	4.253	***	Accept

Note: * *p* < 0.05, *** *p* < 0.001.

## Data Availability

The raw data supporting the conclusions of this article will be made available by the authors, without undue reservation, to any qualified researcher.
